# The mechanisms of influence in intergenerational trauma transmission from mother to baby after the war in Bosnia-Herzegovina: what have we learned since then?

**DOI:** 10.1192/j.eurpsy.2023.431

**Published:** 2023-07-19

**Authors:** K. Le Roch

**Affiliations:** Psychology, Rene Descartes University, Paris, France

## Abstract

**Introduction:**

After the war in Bosnia-Herzegovina that lasted from 1992 to 1995, the populations gradually rebuilt their lives haunted by the spectrum of personal and collective painful souvenirs. Regarding the children who were born after the war, some mothers would rather not talk about it to protect their children from what they might be feeling while others would definetely share what they have experienced to protect their children. Because the intergenerational transmission of war trauma from mother to baby has been poorly explored among populations who recently lived in a warzone, we seeked to highlight the particularity of the interpersonal relations between the mother and her child in connection with maternal psychotrauma, by searching the factors of influence on the functioning of the dyad.

**Objectives:**

The main objective of this study is to determine and conceptualize the modes of transmission of trauma from the study of mother-baby interactions in a Bosnian environment, after the war in Bosnia-Herzegovina.

**Methods:**

In 2003, 40 mothers and their babies aged 2 to 36 months living in Sarajevo were enrolled in the study. Among these mothers, 31 lived in or near the warzone and 9 were either displaced in other areas of the country or were refugees in foreign countries. We assessed the level of severity of post-traumatic stress disorder (PTSD) using the Clinician Administered PTSD Scale (Blake et al, 1998). In order to examine the quality of the mother-child dyads of mothers who lived through the war, we videorecorded a 10-minute free play of 23 dyads in their home environment. Then the interactions were coded using the National Institute of Child Health and Human Development observation grid by two independent raters.

**Results:**

The results showed that all mothers who lived through the war presented post-traumatic symptoms but only half of them showed a PTSD. Videotaped observations of mother-child interactions during playtimes revealed that their interactions are less linked to the mother’s PTSD than to the influence of PTSD on maternal attitudes and thereby extending to those of their infant. They are less sensitive to their children’s signals. They are also more intrusive and detached. Overall, they are more focused on themselves than on their child when they are interacting. As a result, their children are more focused on play and less actively engaged in communicating with their mothers.

**Conclusions:**

The interactions between the mother and her child cover a set of relatively complex processes during which the two partners influence each other. When a mother lives through the war, she will pass on to her child an often painful life story. And thus, it is not only the content but also the way she transmits it that influences how the child receives the objects of the transmission.

**Disclosure of Interest:**

None Declared

